# Clinical and genetic analysis of pseudohypoparathyroidism complicated by hypokalemia: a case report and review of the literature

**DOI:** 10.1186/s12902-022-01011-9

**Published:** 2022-04-11

**Authors:** Shaohan Huang, Yingzi He, Xihua Lin, Shuiya Sun, Fenping Zheng

**Affiliations:** Department of endocrinology, Sir Run Run Shaw Hospital, Zhejiang University School of Medicine, Hangzhou, China

**Keywords:** Pseudohypoparathyroidism, *GNAS* abnormal methylation, Hypokalemia

## Abstract

**Background:**

Pseudohypoparathyroidism (PHP) encompasses a highly heterogenous group of disorders, characterized by parathyroid hormone (PTH) resistance caused by mutations in the *GNAS* gene or other upstream targets. Here, we investigate the characteristics of a female patient diagnosed with PHP complicated with hypokalemia, and her family members.

**Case presentation and gene analysis:**

A 27-year-old female patient occasionally exhibited asymptomatic hypocalcemia and hypokalemia during her pregnancy 1 year ago. Seven months after delivery, she experienced tetany and dysphonia with diarrhea. Tetany symptoms were relieved after intravenous calcium gluconate supplementation and she was then transferred to our Hospital. Laboratory assessments of the patient revealed hypokalemia, hypocalcemia and hyperphosphatemia despite elevated PTH levels. CT scanning of the brain revealed globus pallidus calcification. Possible mutations in *GNAS* and hypokalemia related genes were identified using WES, exon copies of STX16 were analized by MLPA and the methylation status of *GNAS* in three differential methylated regions (DMRs) was analyzed by methylation-specific polymerase chain reaction, followed by confirmation with gene sequencing. The patient was clinically diagnosed with PHP-1b. Loss of methylation in the A/B region and hypermethylation in the NESP55 region were detected. No other mutations in *GNAS* or hypokalemia related genes and no deletions of STX16 exons were detected. A negative family history and abnormal DMRs in *GNAS* led to a diagnosis of sporadic PHP-1b of the patient.

**Conclusions:**

Hypokalemia is a rare disorder associated with PHP-1b. Analysis of genetic and epigenetic mutations can aid in the diagnosis and accurate subtyping of PHP.

**Supplementary Information:**

The online version contains supplementary material available at 10.1186/s12902-022-01011-9.

## Background

Pseudohypoparathyroidism (PHP) encompasses a group of rare and heterogeneous metabolic disorders that share a common feature, namely impairment in various hormone signaling pathways that activate cyclic adenosine monophosphate (cAMP) via the Gsα protein and promote resistance to parathyroid hormone (PTH) [1]. PHP is characterized by hypocalcemia, hyperphosphatemia and elevated serum PTH levels. Some patients also develop physical deformities. Albright hereditary osteodystrophy (AHO) was first described in association with PHP in 1942, by Albright et al. [2]. Characteristics included a round face, brachydactyly, subcutaneous calcifications, short stature, obesity, and mental retardation. Studies have shown that PHP is mainly associated with the *GNAS* gene, which is located on chromosome 20q13 and consists of 13 exons and 12 introns. The *GNAS* gene is an imprinting gene that produces several alternatively spliced transcripts, including Gsα, XLαs and NESP55, as well as A/B (also called 1A) and AS, differential methylated regions (DMRs) of *GNAS*. Such DMRs, comprised of CpG-rich regions of DNA, demonstrate ∼50% methylation because either the maternal or paternal allele is methylated. These regions of parental allele-specific methylation are generally maintained in all somatic tissues, whereas expression is occasionally cell type- or tissue-specific, possibly depending on the availability of specific proteins that are permissive for transcription. A/B, XLαs and AS transcripts are fully expressed in paternal alleles, whereas the NESP55 transcript promoter is fully expressed in maternal alleles and both promoters are methylated on inactive alleles [3–5].

PHP is typically classified as type 1 or type 2. Type 1 is distinguished from type 2 by the abnormal cAMP response in urine by exogenous PTH stimulation [6]. Type 1 PHP can be further subtyped into PHP-1a, PHP-1b and PHP-1c according to the presence or absence of AHO, together with the measurement of Gsα protein activity in peripheral erythrocyte membranes in vitro. PHP-1b, in most case lack the evidence for AHO, is caused by epigenetic changes at one or several DMR within *GNAS*. In terms of the decreased Gsα activity, PTH-1a and PHP-1b can overlap in clinical findings. PHP-1a can sometimes present as a mild phenotype of AHO [7]. Zazo et al. [8] reported methylation defects in patients with AHO (ex. mild brachydactyly and partial resistance to TSH), indicating a complex connection between genetic or epigenetic changes and AHO [9, 10]. Thus, to ensure accurate diagnosis and subtyping of PHP, genetic analysis of mutations in *GNAS* exons, as well as epigenetic modifications, should also be considered. PHP is often complicated by an imbalance of electrolytes. Takatani et al. [11] reported significantly reduced magnesium levels in PHP-1b patients. However, PHP-1b accompanied by persistent hypokalemia without hypomagnesemia has rarely been reported. The present study provides novel insight into PHP-1b.

## Case presentation and gene analysis

**Fig. 1:**
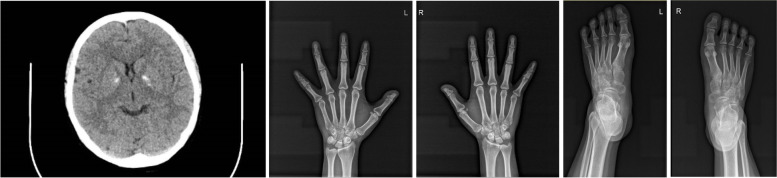
**A** CT scan of the patient’s brain: Calcification of bilateral globus pallidus (arrows). **B** X-ray scan of the patient’s hands: no obvious abnormalities were seen (L: left hand, R: right hand). **C** X-ray scan of patient’s feet: no obvious abnormalities (L: left foot, R: right foot)

In May 2019, a 27-year-old female patient presented with paroxysmal hands tetany and was admitted to the Sir Run Run Shaw Hospital Affiliated to Zhejiang University. The patient occasionally exhibited asymptomatic hypocalcemia and hypokalemia during her pregnancy 1 year ago, upon which she received calcium supplementation. Seven months after delivery, she experienced tetany and dysphonia with diarrhea, which lasted for 2 h. She then presented with hypokalemia, hypocalcemia and high PTH upon admission to the local hospital. Tetany symptoms were relieved after intravenous calcium gluconate supplementation and she was then transferred to our Hospital where a clinical diagnosis of PHP was made based on the laboratory assessments (Table 1). She had hypocalcemia and hyperphosphatemia despite elevated PTH levels, and her 24-h urinary calcium excretion was low. She had hypokalemia with renal potassium loss and elevated direct renin concetraion and aldersterone concentration in plasma, but the blood-gas analysis was normal. The urinary β2 microglobulin levels were increased. The serum magnesium, creatine levels and 25-hydroxyvitamin D3 levels were normal. Thyroid function assement revealed normal thyroid stimulating hormone levels and thyroixine levels with negative thyroid autoantibody. She had normal sex hormone tests including luteinizing hormone, follicle stimulating hormone and estradiol and cortisol rhythms. Computed tomography (CT) scanning of the brain revealed globus pallidus calcification (Fig. 1A). The patient had no features of AHO. X-rays of limbs revealed no brachydactyly (Fig. 1B and C). Her symptoms showed improvement after she received oral calcium and potassium supplementation. Following discussions about the disease, the patient and her family members, including her parents and younger sister, gave written informed consent to participate in a genetic evaluation. All the family members had no AHO features, and their levels of calcium and phosphate, as well as the PTH levels were all normal (Table 2).

**Table 1:** Laboratory results of the patient

Blood results	Patient’s result	Range
Potassium (mmol/L)	3.13	3.5–5.3
Calcium (mmol/L)	1.77	2.11–2.52
Phosphorus (mmol/L)	1.77	0.85–1.51
Alkaline phosphatase (U/L)	155	35–135
Magnesium	0.85	0.75–1.02
Parathyroid hormone (ng/L)	422.20	15–65
Calcitonin (pg/mL)	2.100	0–18
25-hydroxyvitamin D3 (ng/mL)	28.25	20–80
24-h urine calcium (mmol/24 h)	0.7	
Synchornized blood calcium (mmol/L)	1.76	2.11–2.52
24-h urine potassium (mmol/24 h)	40.28	
Synchornized blood potassium (mmol/L)	3.28	3.5–5.3
Urinary β2 microglobulin (mg/L)	1.27	0.1–0.3
Serum creatinine (μmol/L)	56	41–73
Thyroglobulin antibody (TGAb) (IU/ml)	3.75	0–4.11
Thyroperoxidase antibody (TPOAb) (IU/ml)	4.46	0–5.61
Thyroid-stimulating hormone (TSH)(mIU/L)	3.67	0.35–4.94
Free triiodothyronine (FT3) (pg/mL)	3.03	1.71–3.71
Free thyroxine (FT4) (ng/dL)	0.99	0.7–1.48
Plasma aldosterone concentration (PAC) (ng/dL)	31.7	
Direct renin concentration (PRC) (μIU/mL)	69.29	4.4–46.1

**Table 2:** Laboratory results of the family members

	Father	Mother	Sister	Range
K^+^(mmol/L)	4.25	4.33	4.02	3.5–5.3
Ca^2+^(mmol/L)	2.4	2.33	2.43	2.11–2.52
P(mmol/L)	0.85	1.07	1.17	0.85–1.51
Cr (μmol/L)	69	78	68	57–97
AKP (U/L)	108	107	88	35–135
PTH (ng/L)	57.46	62.43	57.5	15–65
TSH (mIU/L)	0.69	2.14	1.97	0.35–4.94
TT4 (μg/dL)	11.64	9.16	10.63	4.87–11.72
FT4 (ng/dL)	1.26	1.03	1.19	0.7–1.48
TPOAb (IU/mL)	1.64	0.51	0.23	0–5.61
TT3 (ng/mL)	1.11	1.18	1.16	0.58–1.59
FT3 (pg/mL)	3.21	3.16	3.28	1.71–3.71
TGAb (IU/mL)	1.51	2.15	1.85	0–4.11

Genomic DNA from the patient and family members was extracted from whole blood leukocytes. Then DNA samples were analyzed by Dian Diagnostics Group Co., Ltd. through Sanger sequencing of the *GNAS* gene, as well as Bartter and Gitelman syndrome-related genes: *SLC12A1, KCNJ1, CLCNKB, BSND, CASR* and *SLC12A3.* Simultaneously using MLPA to detect the exon copies of STX16. However, no pathogenic mutation was found in exons of *GNAS* and Bartter and Gitelman syndrome-related genes by Whole-exons sequencing (WES) analysis. Futhermore, copies of exons of STX16 were nomal. Then, DMRs of *GNAS* were further measured as following method: first, DNA samples were methylated using a EpiTect Plus DNA Bisulfite Kit. Primers targeting DMRs of the *GNAS* gene (NM_000516.4) were designed for PCR amplification (Table 3). The PCR program was as follows: 95 °C for 5 min, 95 °C for 30 s, 60 °C for 30 s, and 72 °C for 30 s. For the first 10 cycles, the annealing temperature of each cycle was reduced by 1 °C; then, the temperature was decreased to 50 °C for 25 cycles (35 cycles in total). A final extension at 72 °C for 10 min was performed before samples were cooled to 4 °C for 10 min. Then, the PCR products were then analyzed by electrophoresis using a 1% agarose gel, sequenced by Qingke Biological Technology Co., Ltd.

**Table 3:** Forward and reverse primers were designed to target the A/B, XLαs and NESP55 regions

Primers	Primer sequence
A/B(M)F	5′-TTCGGCGGGGATATTTAGTC-3′
A/B(M)R	5′-ACAAAAACTCGCTCCAACCG-3′
A/B(U)F	5′-TGGTATTGTGGAGTGGGTTG-3′
A/B(U)R	5′-CCCCACACCAAAACAAAAAC-3’
XLαs(M)F	5′-GTTCGGTTGGGTGTTTTATTTTAC-3’
XLαs(M)R	5′-ATAATTACTCGAACTATTCCCCGAT-3’
XLαs(U)F	5′-GTTTGGTTGGGTGTTTTATTTTATG-3’
XLαs(U)R	5′-ATAATTACTCAAACTATTCCCCAAT-3’
NESP55(M)F	5′-CGTTTTTGTTATTTTTAACGTTCGT-3’
NESP55(M)R	5′-ACAACTCAAAATCTACCTCCTCGTA-3’
NESP55(U)F	5′-TGTTTTTGTTATTTTTAATGTTTGT-3’
NESP55(U)R	5′-ACAACTCAAAATCTACCTCCTCATA-3

*F* Forward primer, *R* Reverse primer, *M* Methylated primer, *U* Unmethylated primer

**Fig. 2:**
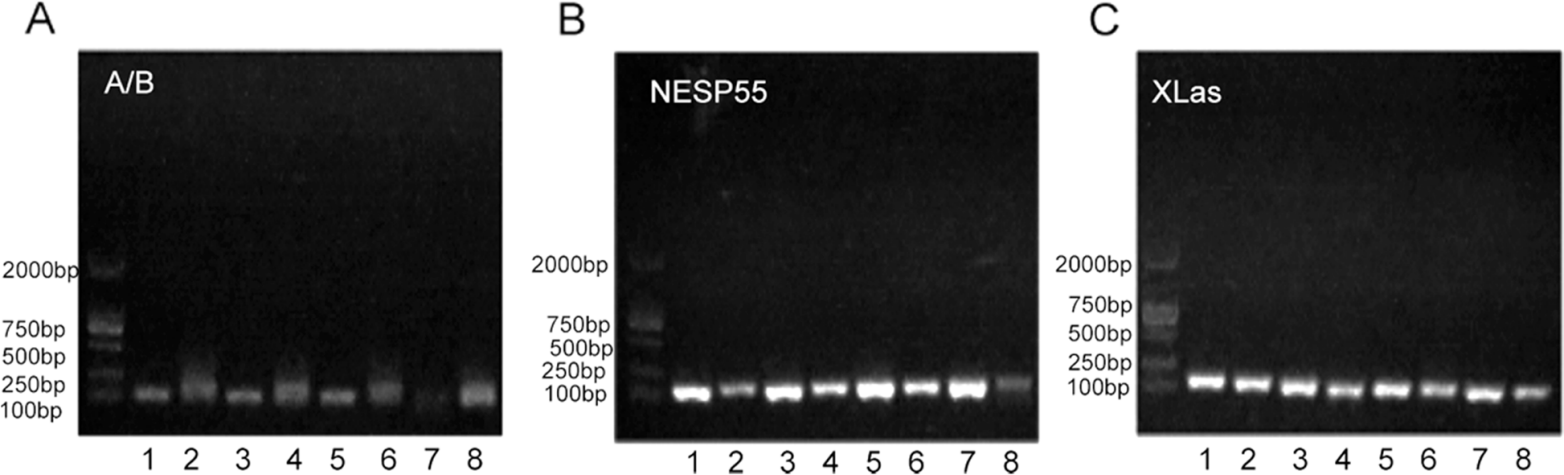
Amplification of the A/B region (**A**), NESP55 region (**B**) and XLαs region (**C**) in the patient and family DNA samples.1: Father: M, 2: Father: U, 3: Mother: M, 4: Mother: U, 5: Younger sister: M, 6: Younger sister: U, 7: Patient: M, 8: Patient: U. M: Methylated primer, U: Unmethylated primer

**Fig. 3:**
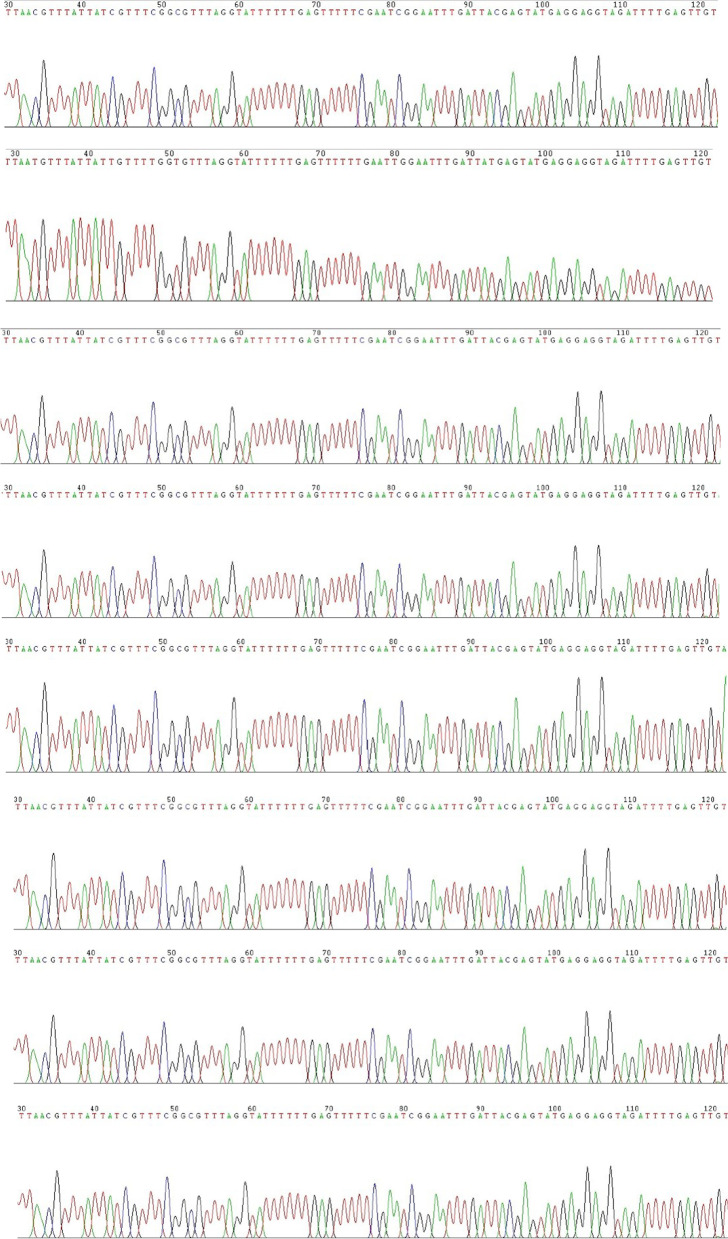
The sequence of the patient DNA amplified using methylated and unmethylated primers targeting the A/B region. M: No change was observed in the amplified sequence of the patient DNA with methylated primers. U: C is converted to T in the amplified sequence of the patient DNA with unmethylated primers (arrows)

In the patient sample, the band indicating the A/B exon was detected only when using unmethylated primers, indicating loss of methylation (LOM) in this region. In addition, hypermethylation in the region of NESP55 was detected using methylated primers (Fig. 2). Methylation-specific PCR involves melting the DNA duplex after bisulfite treatment. If the CpG island of the DNA fragment has not methylated, the cytosine (C) in the sequence is completely converted to uracil (U), and then to thymine (T). If the CpG island has been methylated, this change will not occur. In the A/B region, there was no change of C in the sequence amplified from the patient sample using methylated primers, but C was converted to T when using unmethylated primers (Fig. 3). No similar changes were observed in the A/B region in the samples taken from the patient’s family members (Fig. 3). Sequencing results of the NESP55 and XLαs region were normal both in the patient and in family members.

## Discussion and conclusions

Here, we report a PHP patient with chronic hypocalcemia and hypokalemia. She had no appearance of AHO. A clinical diagnosis of PHP-1b was made initially based on hypocalcemic tetany, hyperphosphatemia with significantly elevated PTH levels, which indicated PTH resistance. The diagnosis was finally diagnosed by epigentic analysis of *GNAS.* CT scanning revealed bilateral calcification of the globus pallidus in the patient, indicating long-duration hypocalcemia and hyperphosphatemia leading to ectopic calcifications within the brain.

It is generally agreed that AHO specifically appears in PHP-1a patients, and PHP-1b patients have no AHO features. However, Levine et al. [9] reported that mild brachydactyly was found in some PHP-1b patients, thus genetic and epigenetic analysis is necessary for diagnosis and accurate subtyping. We did not find any mutation in exons of *GNAS*, but confirmed abnormal methylation status of the DMRs of *GNAS,* which is consistent with the diagnosis of PHP-1b. PHP-1b can be further divided into sporadic or autosomal dominant PHP-1b (AD-PHP-1b) [12].

Studies have indicated that cases of sporadic PHP-1b commonly present with a LOM at exons A/B, XLαs or AS in the *GNAS* gene, and a gain of methylation (GOM) at exon NESP55 [10, 13–15]. Liu et al. [16] proposed that a loss of maternal-specific methylation of exon A/B in PHP-1b leads to PTH resistance due to a tissue-specific loss of Gsα expression. Some cases may show impairment in the maintenance of methylation in early embryos [17]. In most patients diagnosed with AD-PHP-1b, a 3-kb microdeletion of STX16 upstream of *GNAS* results in the removal of exons 4–6 of STX16. This mutation is linked with the LOM observed in the A/B region but has no association to any other exon found in the DMRs of *GNAS*. Therefore, the two subtypes of PHP-1b can be clearly distinguished based on epigenetic factors [17–19]. All the family members of our patient had no AHO features with normal electrolyte and PTH levels. LOM in the A/B region and GOM in the NESP55 region, as well as normal exon copies of STX16 were identified in the patient, which led to a final diagnosis of sporadic PHP-1b when combined with the family history.

Upon admission, the patient exhibited persistent but mild hypokalemia, increased potassium excretion in the urine, and no metabolic alkalosis. Serum levels of magnesium were normal. The urinary levels of β2 microglobulin were increased, as were the plasma concentrations of renin and aldosterone. Taking these findings into consideration, a diagnosis of Bartter syndrome was considered. Previous studies in Japan have reported cases of PHP comorbid with Bartter syndrome, with patients also presenting with hypokalemia and metabolic alkalosis, as well as high plasma concentrations of renin and aldosterone [20]. However, the patient in this study had no pathogenic mutations of genes related to Bartter or Gitelman syndrome. Recently, a case of PhP-1b with hypokalemia was reported [21]. It seems that hypokalemia is more common in PhP-1b. At present, studies aimed at to determining the possible molecular mechanisms of PHP that lead to hypokalemia are ongoing in Japan. It has been suggested that potassium channels within lumen, and peritubular membranes located in the medullary thick ascending limb, play an important role in the circulation of potassium. The Gsα/cAMP/PKA signaling pathway is known to promote the activity of these channels. The kidneys of PHP patients are resistant to PTH, leading to reduced levels of cAMP and downregulation of this signaling pathway. This inhibition poses an obstacle to the recycling of potassium. The mechanism is similar to the hypokalemia caused by Bartter syndrome [22]. On the other hand, it is thought that hypocalcemia alone may lead to the degeneration of renal tubular epithelial cells, which in turn may cause renal tubular dysfunction leading to the disruption of potassium reabsorption [23]. The symptoms such as tetany did not recur after our patient received oral treatment with calcium and potassium supplement.

Our case showed that both mutational analysis of *GNAS* exons and evaluation of *GNAS* imprinting can help diagnose PHP and improve the accuracy of subtyping. We also showed that hypokalemia may be a rare concomitant disorder of PHP-1b and provide new insight into PHP disease.

## Supplementary Information

**Additional file 1.**

**Additional file 2.**

## Data Availability

The datasets of the sequence of DNA amplified using methylated and unmethylated primers targeting the A/B region generated during the current study are available in the NCBI repository, https://www.ncbi.nlm.nih.gov/bioproject/PRJNA820369/. Any additional information is available from the authors upon request.

## Abbreviations

AD-PHP-Ib: Autosomal dominant PHP-Ib
AHO: Albright hereditary osteodystrophy
DMRs: Differentially methylated regions
AS: GNAS antisense
GNAS: Guanine nucleotide-binding protein, a stimulating
GOM: Gain of methylation
Gsα: αlpha-subunit of the stimulatory G protein
GnRH: Gonadotropin releasing hormone
LOM: Loss of methylation
MLPA: Multiplex ligation-dependent probe amplification
MF: Methylated-specific forward primer
MSP: Methylation-specific PCR
MR: Methylated-specific reverse primer
NESP55: Neuroendocrine secretory protein 55
PCR: Polymerase chain reaction
PHP: Pseudohypoparathyroidism
PTH: Parathyroid hormone
sporPHP-Ib: sporadic PHP-Ib
TSH: Thyroid stimulating hormone
UF: Unmethylated-specific forward primer
UR: Unmethylated-specific reverse primer
WES: Whole-exons sequencing
XLαs: Extralarge Gsα
