# Supplementary material for: Clinical and genetic analysis of pseudohypoparathyroidism complicated by hypokalemia: a case report and review of the literature
Source: BMC Endocr Disord. 2022 Apr 11;22:98. doi: 10.1186/s12902-022-01011-9 (PMC9004107; doi:10.1186/s12902-022-01011-9)
Supplement: Supplementary file 1 — Additional file 1. [file 12902_2022_1011_MOESM1_ESM.docx]

Additional files

Additional information

Title of data：Figure1A
Description of data：CT scan of the patient’s brain: Calcification of bilateral globus pallidus (arrows).

Title of data：Figure1B
Description of data：X-ray scan of the patient's hands: no obvious abnormalities were seen (L: left hand, R: right hand).

Title of data：Figure1C
Description of data：X-ray scan of patient's feet: no obvious abnormalities (L: left foot, R: right foot).

Title of data：Figure2ABC
Description of data：Amplification of the A/B region (A), NESP55 region (B) and XLαs region(C) in the patient and family DNA samples.1: Father: M, 2: Father: U, 3: Mother: M, 4: Mother: U, 5: Younger sister: M, 6: Younger sister: U, 7: Patient: M, 8: Patient: U. M: Methylated primer, U: Unmethylated primer.

Title of data：Figure3

Description of data：The sequence of the patient DNA amplified using methylated and unmethylated primers targeting the A/B region. M: No change was observed in the amplified sequence of the patient DNA with methylated primers. U: C is converted to T in the amplified sequence of the patient DNA with unmethylated primers (arrows).
